# Understanding the Dynamics of Gene Regulatory Systems; Characterisation and Clinical Relevance of *cis-*Regulatory Polymorphisms

**DOI:** 10.3390/biology2010064

**Published:** 2013-01-09

**Authors:** Philip Cowie, Ruth Ross, Alasdair MacKenzie

**Affiliations:** School of Medical Sciences, Institute of Medical Sciences, University of Aberdeen, Aberdeen, Scotland, AB25 2ZD, UK; E-Mails: p.cowie@abdn.ac.uk (P.C.); r.ross@abdn.ac.uk (R.R.)

**Keywords:** gene regulation, *cis*-regulatory variation, non-coding DNA, chromatin, signal transduction, drug response stratification, cell specificity, context dependency, ENCODE consortium

## Abstract

Modern genetic analysis has shown that most polymorphisms associated with human disease are non-coding. Much of the functional information contained in the non-coding genome consists of *cis*-regulatory sequences (CRSs) that are required to respond to signal transduction cues that direct cell specific gene expression. It has been hypothesised that many diseases may be due to polymorphisms within CRSs that alter their responses to signal transduction cues. However, identification of CRSs, and the effects of allelic variation on their ability to respond to signal transduction cues, is still at an early stage. In the current review we describe the use of comparative genomics and experimental techniques that allow for the identification of CRSs building on recent advances by the ENCODE consortium. In addition we describe techniques that allow for the analysis of the effects of allelic variation and epigenetic modification on CRS responses to signal transduction cues. Using specific examples we show that the interactions driving these elements are highly complex and the effects of disease associated polymorphisms often subtle. It is clear that gaining an understanding of the functions of CRSs, and how they are affected by SNPs and epigenetic modification, is essential to understanding the genetic basis of human disease and stratification whilst providing novel directions for the development of personalised medicine.

## 1. Introduction

The importance of gene regulation cannot be overstated; the evolution of complex multicellular organisms whose cells possess identical genomes, yet exhibit phenotypic and functional diversity, coincides with the evolution of complex gene regulatory systems capable of controlling differential gene expression [1,2]. Further, multicellular life must have the ability to regulate its transcriptome in response to extracellular signals from the environment, and surrounding cells if it is to develop, adapt and survive. To this end eukaryotes have evolved a repertoire of extracellular signals and receptors which activate diverse signal transduction pathways ultimately resulting in the regulation of specific genes through recruitment of transcription factor (TF) complexes [3]. Central to this process in many genes is the involvement of *cis-*regulatory sequences (CRSs); non-coding functional regions of DNA which mediate TF binding and regulate transcription [4].

Interest in *cis-*regulatory sequences has intensified since the human genome sequence was first mapped [5,6] and subsequently shown to only contain 20,000–25,000 protein coding genes [7]; far fewer than was previously anticipated, leaving ~97% of the genome with no predicted coding function. Consequently, comparative genomics [8,9] has been used to demonstrate that conservation of non-coding DNA regions between evolutionarily divergent species is a powerful tool for the prediction of *cis-*regulatory sequences [10,11,12,13] including promoter and enhancer regions, insulators and locus control regions (reviewed; [14]). More recently, the international consortium ENCODE published a series of papers highlighting that 80.4% of the human genome functions in some form of biological process, and conservative estimates suggest that there may be 4.5 times more functional information within the genome than that which encodes proteins [15].

Given the fundamental role CRSs play in gene regulation, and the necessity for precise regulation to orchestrate correct development and function, it comes as no surprise that variation within CRSs is emerging as a major source of disease susceptibility in human populations [16]. Meta-analysis of multiple genome wide association (GWA) studies [17,18] indicates that 88% of disease-associated single nucleotide polymorphisms (SNPs) lie in intronic or intergenic regions [19]. More specifically, 71% of disease-associated SNPs (including SNPs in linkage disequilibrium) lie in non-coding regulatory regions identified by ENCODE [15]. Hence polymorphisms of non-coding regulatory regions are disproportionately linked to human disease likely through mechanisms involving aberrant gene regulation. In principle, these gene regulation aberrations will not only impact an individual’s susceptibility to disease but also their response to drug treatments as a result of underlying biochemical differences.

A significant challenge for molecular genetics is therefore to: (1) determine the tissue-specific nature of *cis*-regulatory relationships within 3 dimensional paradigms; (2) locate interacting partners of CRSs (3) apply computational and experimental approaches to understand how they function in regulatory networks; (4) evaluate the effect of endogenous CRS variation in the context of cellular signalling and (5) determine the role that CRS variation plays in human disease and drug response stratification.

## 2. The Importance of Non-Coding DNA

**Figure 1:**
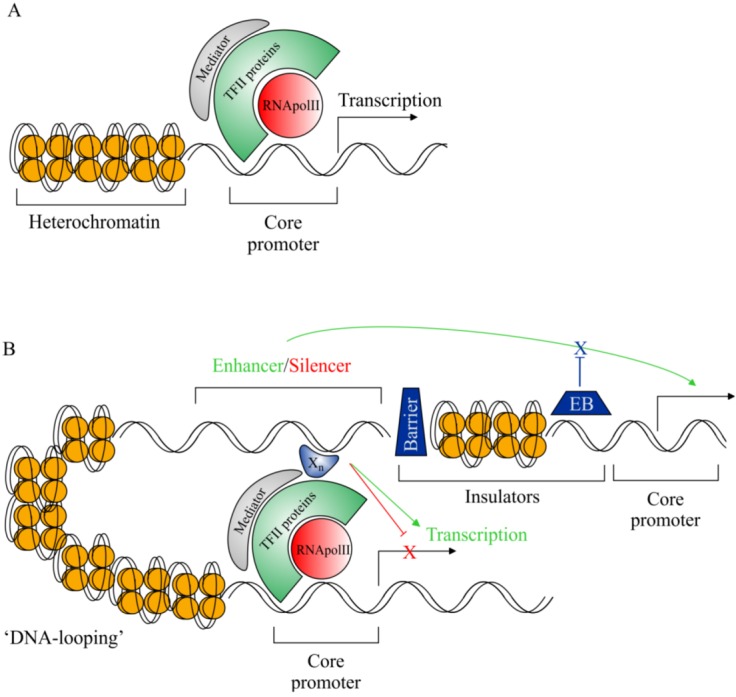
Graphic representation of eukaryotic transcriptional machinery. (**A**) Basal eukaryotic transcriptional machinery; members of the transcription factor II (TFII) family of proteins associate with RNA polymerase II (RNApolII) in an ordered manner to form the pre-initiation complex. The core promoter region, containing transcription factor binding sites (TFBS) and the transcriptional start site, is bound by the pre-initiation complex and RNApolII is directed to begin transcription of target genes. (**B**) *cis-*regulatory DNA sequences modulating eukaryotic transcription. Distant *cis*-regulatory sequences (CRSs), such as enhancers and silencers (located up to 1Mbp from the target promoter), associate with additional TFs (X_n_) and form indirect interactions with the target promoter. Subsequently, transcriptional outputs are modified depending on the nature of the associated CRS; increases in transcript quantity (enhancer function—green arrows) or reduction/abolition of transcription (silencer function—red T-bars). In order for enhancer/silencer sequences to interact with target promoters DNA must be modified to “loop out” the interspaced DNA. Other recognised classes of regulatory sequences include insulators: Barrier-form insulators prevent chromatin condensation from repressing active regulatory regions setting up regulatory boundaries; Enhancer-blocking (EB) insulators maintain the specificity of CRS interactions by blocking regulatory sequences from impinging on neighbouring genes. Finally, locus control regions are described as regions containing multiple CRSs, they function in concert to confer correct temporal and/or spatial specificity of the target gene.

As a prerequisite to understanding developments within the field of CRS research we have outlined some basic aspects of eukaryotic transcription with respect to transcriptional machinery and *cis-*regulatory functions (Figure 1). To appreciate the value of studying non-coding DNA, and its role in gene regulation, we must evaluate its importance with respect to evolution and development and determine its pathological potential.

### 2.1. cis-Regulatory Sequences Have Shaped Human Evolution and Development

A critical feature of CRSs is the modular nature by which they regulate gene expression [20]. Thus tissue-specific (spatial) and developmental stage-specific (temporal) gene expression can be controlled by specific CRS-mediated TF-complex binding. The apolipoprotein E (*APOE*) locus is a well characterised example of a gene that is regulated by multiple flanking CRSs that direct differential expression to liver cells [21,22] or skin cells [23,24], or astrocytes, macrophages and adipocytes [24,25]. Consequently, the effects of mutations in CRSs can be limited to particular cell types or developmental stages making them less pleiotropic than coding mutations. The relative lack of pleiotrophism makes CRSs strong candidates for driving evolution through mutation as well as inducing susceptibility to late onset disease. For example a CRS SNP located upstream of the *DARC* promoter, which codes a human receptor important for the reception of immune system signals [26,27,28], abolishes expression of the receptor in erythrocytes [29,30]. This SNP confers complete resistance to malaria [31,32] by preventing *Plasmodium spp.* parasites entry to erythrocytes due to the lack of the *DARC*-coded receptor [33,34]. Importantly, the SNP has little or no deleterious effects in other *DARC* expression domains. Another example is *HACNS1*, a highly conserved non-coding sequence, which has been identified to contain human-specific polymorphisms that result in the differential limb patterning observed between humans and non-human primates [35].

### 2.2. cis-Regulatory Sequences are Implicated in Human Pathologies

With respect to human pathologies it was shown that a non-coding regulatory SNP located near the α-globin gene cluster creates a new TF consensus sequence for GATA-1 augmenting the activation of the gene cluster and causing Thalassemia in affected individuals [36]. Further, very recent data concerning the transcription factor 7-like (*TCF7L2*) locus has utilised the results of GWA studies, identifying variation within the *TCF7L2* intronic regions as highly associated with risk of type 2 diabetes, and shown that the associated variation is located within a *cis-*regulatory region [37]. Moreover it has been discovered that Hirschsprung disease risk is associated with variation within an enhancer region of the receptor tyrosine kinase *RET* [38,39]. While coding mutations in *RET* were causative in a small portion of cases the authors also found that variation within a CRS of *RET* intron 1 resulted in a significant decrease in *RET* expression [38,39].

### 2.3. Rationale for cis-Regulatory Sequence Research

It is clear from these examples that CRSs play a vital role in evolution, development and human disease, indeed preeminent conjectures concerning the importance of CRSs to evolution and development through gene regulation were made ~40 years ago by Jacob and Monod [40], Britten and Davidson [41,42] and King and Wilson [43]. However, despite the wealth of evidence which has been mounting in recent years CRSs remain relatively poorly understood. This is due in part to decades of exon-focused research, which by comparison has more easily definable and testable entities. Intriguingly, computational analysis has shown that 87% of the conserved genome between humans and mice (>70% identity over 100 bp) is non-coding which highlights the potentially massive pool of unexamined functional DNA present within the genome [44]. One of the major challenges to examining CRSs is their identification and publication of the human genome sequence [5,6] has proved enormously helpful in addressing this issue. Moreover the collaborative efforts of the ENCODE project has marked a huge step towards elucidating the functional regulatory landscape of the human genome through systematic CRS identification using a number of well characterised computational and experimental paradigms which we have summarised below [15].

## 3. *cis-*-Regulatory Sequence Identification—Comparative Genomics

Comparative genomics has emerged as a powerful tool for the discovery of CRSs and relies on the basic principle that regulatory functional sequences are under purifying selection and cross-species sequence comparisons can highlight this conservation. It is important to note that, while many CRSs regulate target gene expression through TF binding and recruitment to promoters, predicted TF binding motifs do not represent reliable candidate sequence motifs for the identification of CRSs due to their high degeneracy and wide-spread distribution in the genome. Instead we may broadly consider two approaches assessing genome-wide sequence conservation: evolutionary distant species comparisons and evolutionarily related species comparisons.

### 3.1. Evolutionary Distant Species Comparisons

In the first case, the availability of genome sequences from birds, fish and reptiles allow researchers to identify putative CRSs with functions critical to vertebrate development by way of pair-wise comparison to mammalian genomes. This approach has been highly successful for identifying CRSs, even prior to the availability of genome sequences for so many vertebrates [45], such as those involved in the tissue-specific expression of embryogenesis genes related to: cardiac development [46]; limb patterning [13,47,48] and brain development [13,48,49]. Indeed a common feature of CRSs identified by this method is that they are non-randomly located in gene deserts [12,50] adjacent to genes with developmental functions [49].

Unquestionable then is the potential importance of distant comparative approaches, clearly capable of locating vertebrate developmental gene-related CRSs, but there are a number of important caveats to consider. Firstly, altering the parameters of this strategy has been shown to cause estimations of CRS numbers to vary between 1,400 [49] and 5,700 [51], suggesting that the method is insensitive and misses many CRSs since these estimations are an order of magnitude lower than the predicted number of human genes [7]. Additionally, such “deep” conservation is likely to be the result of a shared biological process between the species under comparison; hence this method is unable to identify CRSs involved in processes which evolved subsequent to the divergence of the species in question. Finally, if such comparisons are used between less divergent species such as human-rodent the relaxed parameters (>70% identity over 100 bp) will throw up large numbers of false positive results.

### 3.2. Evolutionary Related Species Comparisons

In the second case, researchers can identify CRSs more likely related to higher vertebrate health by comparing less distant species with more stringent conservations parameters. Specifically, typical conservation parameters between human-chicken or human-frog comparisons are >70% identity over 100 bp. However Bejerano *et al*., (2004) explored the use of human-rodent comparisons at parameters of 100% identity over 200 bp [52]. Unsurprisingly, they found a smaller set of putative CRSs as compared to Woolfe *et al*., (250 [52] and 1,400 [49] respectively), however investigation of some of these “ultra-conserved” sequences has proved, in principle, that the method is capable of identifying modulators of gene transcription [48,53,54]. Interestingly, the method was further assessed in combination with human-fugu comparisons, whereby the authors were able to predict enhancer activity of sequences very successfully (~60% of identified sequences showed enhancer capacity) by coupling “deep” conservation (human-fugu) with ultra-conservation (human-rodent, described above) [13]. However, subsequent investigation into ultra-conservation comparisons has lead some researchers to conclude that overall sequence conservation, as opposed to ultra-conservation, is a good predictor of CRSs functionality [55].

Consequently, ultra-conservation comparison techniques do suffer as a product of their design; they are likely to identify only small subsets of CRSs, and not only miss numerous other CRSs but also cannot be utilised as a large-scale prediction method [11]. Further, the parameters required to fulfil the “ultra-conservation” label mean that many predicted CRSs are also identified by evolutionary divergent comparisons [11]. Likely, even with the manifestation of well characterised highly accurate computation models to predict CRSs, we must acknowledge that computational data alone cannot provide extensive evidence as to biological function. Consequently, parallel experimental approaches have been developed to complement computational prediction of CRSs to good effect.

## 4. *cis-*-Regulatory Sequence Identification—Experimental Approaches

In response to the stated drawbacks of computational conservation-based CRS prediction methods, well developed strategies now exist which allow researchers to identify CRSs in a conservation-independent manner (reviewed [56]). One particular reason for this is the observation that ~50% of experimentally validated CRSs do not show sequence conservation [57], and, depending on the tissue type under investigation, enhancers can been significantly non-conserved [58].

### 4.1. Transcriptional Associations: Chromatin Immunoprecipitation Techniques

A number of the experimental paradigms for CRS identification originate from the exploitation of an indirect physical association between the CRS and its target promoter via TF-complexes and transcriptional co-activators such as p300 [14,59]. Researchers begin determining these interactions by cross-linking chromatin with formaldehyde, capturing endogenous DNA-protein interactions within the nucleus, and subsequently shearing it into smaller pieces by sonication or enzymatic digest. Samples are enriched for DNA showing an association with specific TF’s, co-activators or histone-modifications associated with enhancers (e.g., H3K4me1) or silencers by immunoprecipitation with antibodies specific to the TF, co-activator or histone-modification. The principle technique is called chromatin immunoprecipitation (ChIP), and the resultant enriched samples can be analysed by hybridisation to microarrays (ChIP-chip) [60,61] or by deep sequencing the entire enriched DNA sample (ChIP-seq) [62,63]. Results are analysed for DNA sequences which are over represented in the enriched samples, demonstrating that they are likely associated with TF’s and/or co-activators and therefore involved in transcriptional regulation. This method can also be used on restricted cell populations by initially micro-dissecting specific tissue regions, ChIP results then provide an immediate indication of the tissue-specific activity of identified CRSs [64].

### 4.2. Active Chromatin Signatures: DNaseI Hypersensitivity and Formaldehyde-Assisted Identification of Regulatory Elements

Another approach to discovering CRSs employs the fact that functional non-coding sequences are associated with “active” chromatin conformations, induced through TF binding, making these stretches of DNA more sensitive to DNase I activity [65]. DNase I hypersensitivity (DHS) approaches can again be combined with microarrays or deep sequencing to identify regions of DNA with an “open” chromatin structure indicative of TF binding and presumed regulatory potential [66,67]. Of particular interest, this technique is capable of detecting hypersensitivity differences which result from polymorphisms within the genetic code, highlighting the potential for polymorphic variation in CRSs to impact gene regulation and by extension disease [68]. Further, DHS sites are known to be enriched for non-coding disease-associated genetic variants and commonly map to disease-associated loci [69]. Consequently, DHS data can be highly predictive of disease-associated regulatory networks including causative CRSs and interacting proteins [69,70]. FAIRE (formaldehyde-assisted identification of regulatory elements) is similar to the DNase I hypersensitivity technique, in that it exploits open chromatin’s susceptibility to mechanical shearing after formaldehyde cross-linking to non-selectively identify functional regulatory DNA regions [71]. Both of these methods can provide researchers with fast, cost effective results. Combined with well organised comparative genomic analysis CRSs can often be inferred providing a reliable basis for further study.

### 4.3. Chromosome Interactions: Chromosome Conformation Capture Strategies

The above techniques identify either DNA which associates with transcriptional regulatory proteins (ChIP) or DNA which is putatively active in the binding of transcriptional regulatory proteins (DNase, FAIRE), but neither is able to remote chromatin interactions nor do they provide information relating to the 3-dimensional structure of the genome. Development of chromosome conformation capture (3C) [72], and derived techniques (4C, 5C and Hi-C [73] (see [74] and [75] for review)), overcome this hurdle on the premise that CRSs and promoters must indirectly interact across large regions of the genome. A consequence of these long distance interactions is that, following cross-linking and shearing, DNA can be covalently ligated to sequences in close 3-dimensional proximity (proximity ligation). The experimental output then identifies interactions between DNA sequences, which may normally be separated by up to 1 Mb, being sequenced together more frequently as a result of a 3D chromatin interaction. A drawback of 3C, 4C and 5C is that they are all biased towards a particular locus, or set of loci, under investigation.

Conversely, Hi-C is both genome-wide and unbiased in its identification of long distance chromatin interactions; by incorporating biotinylated residues into the fragment ends after digestion of cross-linked DNA streptavidin can be used to select for sequences in close proximity which are subsequently analysed [73]. Further advancements towards the functional annotation of the genome have resulted in the development of the technique ChIA-PET (chromatin interaction analysis by paired-end tag sequencing) [76,77]. Similar in methodology to Hi-C, but requiring an interacting protein for sample enrichment by immunoprecipitation before proximity ligation, ChIA-PET is seen as a promising alternative to ChIP-Seq since it is capable of identifying both TFBSs and chromatin structure within purified sequences [77,78].

### 4.4. Towards a Map of the Genome’s Regulatory Landscape: The ENCODE Consortium

The ENCODE consortium represents an international project aimed at identifying all the functional elements in the human genome using a combination of computational and experimental approaches [15] (some of which are outlined above). Data generated by the project is available on the UCSC genome website [79,80]; customisable tracks can be selected to view chromatin modification signatures, DNase I hypersensitivity, FAIRE analysis, TF binding sites, transcriptional start sites and DNA methylation patterns for particular genomic regions within a number of different cell type. Consequently, ENCODE data is likely to represent the starting point for the majority of CRS investigations of the future; a vast database of the regulatory landscape of the genome will provide researchers with immediate indications of the regulatory capacity of selected regions. Further, work in progress by ENCODE to complete genome wide chromosome conformation maps will provide researchers with invaluable insights into long distance DNA sequences interactions.

However, we must highlight some caveats of ENCODE’s three tiered cell type strategy [15]. The exclusion of many important primary cell types, such as neuronal cells, has undoubtedly resulted in many CRSs going undetected due to both the context dependent nature of CRSs and their inducibility by cellular signalling events (see: *A question of specificity?* for more information). This ultimately means that while ENCODE data at UCSC will serve as a platform for much CRS research the lack of positive functional information for many highly conserved sequences does not yet persuasively indicate that they are not regulatory but that the particular cell types or specific stimuli used to ascribe functionality have yet to be ascertained.

## 5. Analysis of *cis-*Regulatory Sequences

**Figure 2:**
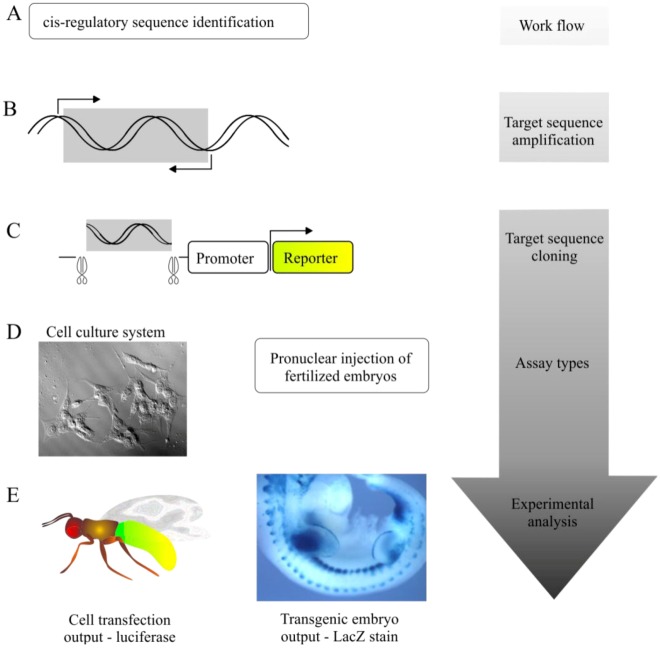
General experimental workflow of *cis-*regulatory sequence studies. (**A**) Numerous well characterised methods for CRS identification exist including computational and experimental approaches (described in main text). (**B**) Identified target sequences (boxed—grey) are reliably amplified via polymerase chain reaction (PCR) using specific primers (arrows). (**C**) Target sequences (putative CRSs) are cloned into a variety of reporter plasmid constructs, including luciferase, *LacZ* and fluorescent protein derivatives (e.g., *GFP*). Typically reporter plasmids are sequenced to ensure sequence integrity. (**D**) Reporter plasmids may be introduced to cell culture-type systems by transfection or into animal embryos by cytoplasmic or pronuclear injection. (**E**) Depending on the assay type a number of experimental outputs are obtainable: cell culture assays can provide quantitative analysis of target CRSs via luminosity readings (e.g., luciferase) and are particularly useful for pharmacological studies (see Figure 3); animal/embryo studies can provide qualitative explanations of where and when the target CRS is active during development.

Two standard approaches used to evaluate putative CRSs are transgenic animal-based reporter gene assays and cell-based reporter gene assays. By providing qualitative and quantitative information (respectively) about CRSs of interest these techniques are widely used in the confirmation of putative regulatory sequences. A schematic representation of CRS research workflow summarises how Section 3, Section 4 and Section 5 are commonly implemented (Figure 2).

### 5.1. Transgenic Animal Reporter Assays

Using analysis of transgenic animals the CRS of interest is typically cloned upstream of a reporter gene such as *LacZ* [81] or GFP, and the resultant construct is injected into fertilized animal embryos typically derived from species such as zebrafish, Xenopus, chicken or mouse. Subsequently, animals containing the construct are assessed for β-galactosidase activity via X-Gal staining or *GFP* expression with fluorescent microscopes. This method provides the chance to assess the ability of the CRS of interest to drive tissue-specific expression of the reporter gene; a central requirement of CRSs in gene regulation.

Transgenic analysis is considered by many researchers to represent the “gold standard” for confirming the tissue specificity of a candidate CRS. A number of hugely successful examples of its use exist [13,48,49,55], in particular Pennacchio and colleagues examined 167 putative CRSs, identified through comparative genomics, and established that 45% of the candidate sequences supported tissue specific expression of *LacZ* in developing mouse embryos [13]. Indeed the majority of deeply conserved CRSs identified to date function in early development [35], and consequently *LacZ* expression is often assessed in embryonic mice [13]. Within our lab CRSs have also been tested for tissue-specific expression in adult mice where our focus relates to their impact in adult neuronal gene regulation as opposed to developmental programmes [82].

Transgenic animal reporter assays alone are not sufficient to confirm the identity of a target sequence as a specific regulator of the proposed target gene. Subsequent in-situ hybridisation or immunohistological staining are required to demonstrate that putative CRS-driven *LacZ* expression co-localises with the endogenous transcript or endogenous protein. Further it is noteworthy that pronuclear injection creates a random insertion of reporter constructs, consequently at least 2 different transgenic lines with corroborating expression patterns are required.

### 5.2. Cell-Based Reporter Gene Assays

In addition to qualitative cell specific analysis it is useful to analyse the effects of SNPs or signal transduction cues on the quantitative activity of candidate CRSs. Putative CRSs are typically PCR amplified and cloned into reporter constructs, upstream of quantifiable reporter genes such as firefly luciferase. These constructs are then transfected into transformed cell lines or primary cell cultures. This method ultimately determines whether the CRS of interest is capable of eliciting a significant effect on the expression of the reporter gene, indicating its potential to function in gene regulation or to determine polymorphic effects.

We have used primary cell-based reporter gene assays to establish the presence of a highly conserved CRS (BE5.2) which functions as a silencer of the brain derived neurotropic factor (*BDNF*) promoter IV that plays a role in modulating mood [83]. Further, the quantitative nature of this method has been employed by our group to analyse the impact of allelic variation on CRS function; we have demonstrated significant allele-dependent changes in the activity of the *galanin* gene enhancer (GAL5.1) in primary hypothalamic neurons using luciferase reporter assays [82].

## 6. Beyond Identification: *cis*-Regulatory Sequence Characterisation

**Figure 3:**
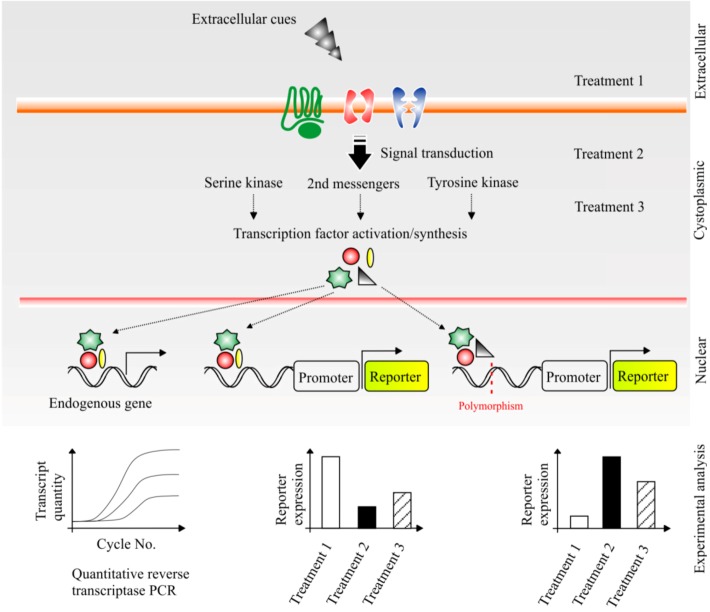
Characterisation of *cis-*regulatory sequences.

CRS characterisation studies are becoming increasingly pertinent in the wake of large scale, high-throughput, genome-wide identification projects (e.g., ENCODE). Vast CRS identification, even when coupled to the aforementioned methodologies, falls short of characterising the intricate signal transduction events which control CRS function. A molecular-level understanding of CRS functions is therefore essential if we hope to exploit them clinically and understand how regulatory polymorphisms impact susceptibility to many common human pathologies. The logic of CRS characterisation studies by pharmacological perturbations (as discussed below) is graphically represented (Figure 3).

### 6.1. Dissecting the Impact of Cellular Signalling

Due to its quantitative output cell-based reporter gene assays provide a means to investigate the cellular systems that modulate the activity of a given CRS through the manipulation of intracellular transduction pathways or ligand-receptor interactions by pharmacological means. The function of CRSs depends on the availability and binding of TF’s and co-activators [4], TF’s are subject to regulation though mechanisms such as extracellular receptor activation, cytoplasmic serine kinase activation and intracellular proteolysis activity [84]. Consequently, cell cultures may be treated with a host of pharmacological agents to elucidate the precise biochemical requirements for CRS-mediated gene regulation. For example, we have previously demonstrated the ability of GAL5.1 to respond to PKC activation [82] and MAPkinase signalling as a necessary cue to the activation of a CRS contained within intron 2 of the CNR1 gene [85]. Similar work has been conducted by the Barolo laboratory as they set about defining the biochemical pathways which regulate the *Drosophila sparkling (spa)* enhancer [86]. Research of this nature is required to define the parameters of CRS function, without knowing the precise events which precede the involvement of a CRS in gene regulation we cannot begin to define their role in disease or produce clinical strategies based on their perturbation.

It is important to determine the relevance of pharmacological CRS manipulation to endogenous gene expression by assessing the effects of these pharmacological agents on the endogenous mRNA levels in parallel using quantitative reverse transcriptase PCR (qrtPCR). This combination of luciferase reporter gene assay and qrtPCR strengthens the argument for a CRS’s capacity to regulate target gene expression. For example using qrtPCR we demonstrated the induction of the *TAC1* gene in primary dorsal root ganglia (DRG) cells by MAPkinase agonism or noxious stimulation by capsaicin. However, as assessed by luciferase reporter assay the *TAC1* promoter alone was unable to respond to these stimuli. Only by combining the *TAC1* promoter with a remote and highly conserved enhancer region called ECR2 could we induce a response from the *TAC1* promoter that was consistent with the response of the endogenous *TAC1* gene. This provides evidence of a requirement for enhancer-promoter synergy at the *TAC1* locus within DRG neurons following noxious induction [87,88].

Rapid development of CRS identification methods and collaborative efforts by the ENCODE consortium have placed an increasing emphasis on the characterisation of newly identified CRSs. Our schematic (Figure 3) shows the layers of a eukaryotic cell (from the extracellular to the nuclear) depicting a simplified cascade of cellular events from: extracellular cues binding to/transporting through cellular receptors; to intracellular transduction pathways; culminating in the production/activation of TFs and ultimately modulating gene transcription accordingly.

Using the previously discussed cell culture assays we highlight how pharmacological treatments aimed at specific cellular processes can potentially alter the activity of a CRS under investigation. For example, in the middle case treatment 2 has defined that the CRS in question is regulated by a particular signal transduction event. Further analysis would eventually determine the specific cellular conditions which precede the recruitment of this CRS to transcription of its target gene. Indeed, this scheme also highlights the potential of such an experimental paradigm to explore the impact of CRS polymorphisms (red line) on gene regulation. In the final case (right) the CRS polymorphism has altered the expression profile regulated by the CRS and perturbation with treatment 2 is now non-effective, a finding which may have clinical implications for individuals with this polymorphism (see: *cis-Regulatory Sequence Variation and Drug Response Stratification*). Finally, the first case (left) highlights the need for this experimental paradigm to include qrtPCR analysis in order to qualify that such changes in reporter gene quantities (either by treatments or by polymorphisms) are corroborated by changes in endogenous transcript quantity of the target gene. Demonstration of alterations in the endogenous transcript quantity indicate the potential for alterations in biochemical events to be associated with the target genes product.

### 6.2. Embryonic Stem Cell Targeting

Despite high financial and time costs, embryonic stem cell targeting studies in mice are required to allow a full analysis of the role of CRSs in development and disease. Employing well defined strategies to knock-in or knock-out CRSs of interest, through the use of Cre-lox or Flp systems [89,90,91,92,93], researchers can define the effects of CRSs, and their polymorphisms on endogenous genes in an *in vivo* system that would be difficult to detect using the previously mentioned primary cell or transgenic strategies. In particular, the developmental role of a CRS may be assessed by knocking it out and analysing resultant changes in body plan, organ development or neuronal patterning. It is worth noting, however, that to date most CRSs are recognised as having modest effects on gene expression and therefore stable transgenic mouse models may only be used when the analysis of the effects of a SNP on CRS function is compelling and has been exhausted by the means described previously.

### 6.3. A Question of Specificity?

To date the majority of CRS studies utilising reporter constructs are conducted using exogenous promoters, and the use of transformed cell lines during analysis by reporter assay. Thus, a seriously underestimated but critical property of CRSs; namely, specificity in terms of promoter specificity and cell-type specificity is being overlooked in these cases.

The principle behind CRS-promoter specificity lies in the fact that CRSs may be located within or beyond neighbouring genes therefore the interaction (e.g., CRS-promoter) that takes place during CRS-mediated transcription relies on the CRS preferentially recognising its specific promoters. Indeed, there are examples of this phenomenon whereby the enhancer required to drive the expression of the *Sonic hedgehog (Shh)* gene in the developing limb bud is found in the intron of a gene lying 1 Mb from the *Shh* locus, called *Lmbr1*, which is also unaffected by its activity [94]. In addition, regulatory elements functioning in trans such as those found in *Drosophila* olfactory receptor genes serve as further evidence of this principle [95]. Whether CRS-promoter interactions are controlled and maintained by levels of chromatin flexibility [96], chromosomal location with the nucleus [97,98,99,100], the interaction of TFs and chromatin remodelling complexes [100], or perhaps a combination of these and undiscovered mechanisms does not alter the principle that CRS-promoter interactions must be specific for the appropriate regulation of their associated genes.

CRS specificity to particular cell types is well documented and a defining feature of their mode of action. Hence experimental approaches aimed at defining the impact of a CRS and/or endogenous CRS variation should also consider the impact that different cell types may have on the ability of the chosen CRS to function accurately. Both ECR1 of *TAC1* [10] and GAL5.1 of the *Galanin* gene [82] exhibit extreme cell-type dependent activity where they are only able to support reporter gene expression in a tiny subset of hypothalamic and amygdala and PVN (paraventricular nucleus) cells respectively [82]; representing a very small fraction of the total cells found within the animal. With this in mind it is essential that CRS characterisation studies include paradigms that most accurately reflect the expression of endogenous candidate genes in order to develop faithful models of CRS-mediated gene regulation. Indeed, many of the reports of non-functionality of highly conserved sequences in the existing literature may stem from a failure to analyse these sequences within an appropriate *in vivo* or primary cell-derived model system in which the appropriate cellular components are active.

## 7. Novel Considerations of *cis*-Regulatory Sequence Polymorphic Variation

### 7.1. cis-Regulatory Sequence Variation and Drug Response Stratification

Variation in drug response within the human population represents an important barrier to clinical drug development by an increasingly pressured pharmaceutical industry. Referred to as drug response stratification, the outcome is often rejection of the drug based on a lack of a significantly positive or unpredictable response. We propose that CRS variation may be a major causative or contributing factor to drug response stratification. Firstly, consider that the effect on any drug is reliant on its perturbation of a targeted biochemical process or of a receptor function. Modulation of receptor function results in alterations of downstream signal transduction systems that, in turn changes gene expression through CRS activation. Changes in the activity of these CRS, as a result of polymorphic or epigenetic variation, may have important consequences for the downstream effects of these drugs thus contributing to drug response stratification. Indeed, research has indicated that stratified responses to glucocorticoid treatments can result from *cis-*regulatory polymorphisms located near glucocorticoid target genes [101]. Further, non-coding SNPs have been identified which significantly inpact the IC_50_ values and cytotoxicity of chemotherapeutic agents highlighting the potential for such SNPs to be used as markers for predicting drug responses. Characterisation of human genome variation may therefore allow genetic screening to determine the likelihood of a positive/negative drug response in advance of clinical trials. Implementation of this strategy will rely on detailed characterisation of CRSs and their variation in part by the techniques described above which are designed to dissect the precise biochemical events associated with CRS-mediated gene regulation.

### 7.2. Genetic and Epigenetic Interaction within CRSs and Disease Susceptibility

DNA methylation, the addition of methyl groups to CpG dinucleotides in the genomic sequence, is a heritable form of epigenetic gene regulation vital to cellular homeostasis and development [102]. The presence or absence of the methyl group has been shown to be affected by early life cues such as starvation or stress, and directly prevents TF-DNA binding thereby altering gene transcription. Furthermore, DNA methylation aberrations are associated with human disease [103]. If we consider this process with respect to CRSs that are critical to gene regulation, it is not unreasonable to conclude that CRSs methylation plays an important role in contributing to human pathologies. For example, it has been shown that methylation of a CRS involved in arginine vasopressin (AVP) gene expression can be altered by early life stress. This results in aberrant hormone secretion leading to changes in passive stress coping and memory [104]. We have also detected allelic variants within the GAL5.1 enhancer which renders it susceptible to DNA methylation through the introduction of a CpG sequence [82]. By contrast, analysis of the ECR1 sequence within *CNR1* intron 2 shows the presence of an allelic variant that confers resistance to DNA methylation [85]. Considering the role that the *Galanin* and *CNR1* genes play in appetite, mood and inflammatory pain these examples suggest the presence of an interplay between genetic and epigenetic variation within CRSs that may have an important baring on our future ability to understand disease susceptibility.
